# Capsaicin protects against septic acute liver injury by attenuation of apoptosis and mitochondrial dysfunction

**DOI:** 10.1016/j.heliyon.2023.e14205

**Published:** 2023-03-01

**Authors:** Atefeh Ghorbanpour, Sepide Salari, Tourandokht Baluchnejadmojarad, Mehrdad Roghani

**Affiliations:** a School of Medicine, Shahed University, Tehran, Iran; b Department of Physiology, School of Medicine, Iran University of Medical Sciences, Tehran, Iran; c Neurophysiology Research Center, Shahed University, Tehran, Iran

**Keywords:** Acute liver injury, Lipopolysaccharide, Capsaicin, Apoptosis, Mitochondrial dysfunction

## Abstract

Capsaicin is the main pungent bioactive constituent in red chili with promising therapeutic properties due to its anti-oxidative and anti-inflammatory effects. No evidence exists on the beneficial effect of capsaicin on apoptosis and mitochondrial function in acute liver injury (ALI) under septic conditions. For inducing septic ALI, lipopolysaccharide (LPS, 50 μg/kg) and d-galactose (D-Gal, 400 mg/kg) was intraperitoneally injected and capsaicin was given orally at 5 or 20 mg/kg. Functional markers of liver function and mitochondrial dysfunction were determined as well as hepatic assessment of apoptotic, oxidative, and inflammatory factors. Capsaicin at the higher dose appropriately decreased serum level of alanine aminotransferase (ALT) and aspartate aminotransferase (AST) in addition to reducing hepatic level of malondialdehyde (MDA), reactive oxygen species (ROS), nitrite, NF-kB, TLR4, IL-1β, TNF-α, caspase 3, DNA fragmentation and boosting sirtuin 1, Nrf2, superoxide dismutase (SOD) activity, and heme oxygenase (HO-1). These beneficial effects of capsaicin were associated with reversal and/or improvement of gene expression for pro-apoptotic Bax, anti-apoptotic Bcl2, mitochondrial and metabolic regulators PGC-1α, sirtuin 1, and AMPK, and inflammation-associated factors. Additionally, capsaicin exerted a hepatoprotective effect, as revealed by its reduction of liver histopathological changes. These findings evidently indicate hepatoprotective property of capsaicin under septic conditions that can be attributed to its down-regulation of oxidative and inflammatory processes besides its potential to attenuate mitochondrial dysfunction and apoptosis.

## Graphical abstract

**Figure:**
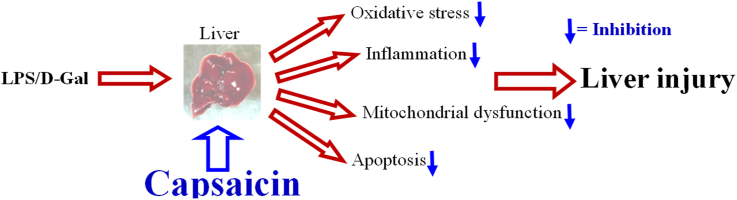

## 1 Introduction

Acute liver injury (ALI) and ensuing hepatic failure is recognized a severe health complication worldwide with high incidence of morbidity and mortality [1]. ALI occurs following exposing to numerous damaging agents such as alcohol, lipopolysaccharide (LPS), carbon tetrachloride (CCL4), and acetaminophen [2,3]. The underlying mechanisms proposed for the development of ALI include multiple factors comprising excessive generation of reactive oxygen species [4], unmanaged inflammation [5,6] and dysregulated apoptotic process [7]. Exposure of d-galactosamine (D-Gal)-sensitized rodents to LPS causes critical liver injury that is usually used for modeling ALI [8,9]. LPS challenge is associated with derangement of mitochondrial biogenesis and oxidative metabolism, leading to mitochondrial dysfunction and induction of liver injury in septic conditions [10].

Natural products with potential to ameliorate oxidative stress and inflammation may be of potential therapeutic benefit to control and prevent ALI [11]. Capsaicin (8-methyl-N-vanillyl-trans-6-nonenamide) is the main pungent bioactive constituent in red chili in the genus Capsicum that has been suggested as a promising therapeutic agent [12]. It possesses some curative effects for the treatment of arthritis, diabetic neuropathy, gastric lesions, and cardiac excitability [13,14]. Capsaicin has also shown potent anti-oxidative effects which is independent of TRPV1 receptor activation [15]. Additionally, capsaicin could exert a hepatoprotective effect against concanavalin A-induced hepatic damage through ameliorating oxidative and inflammatory events [16]. Moreover, protective effect of capsaicin against 2,3,7,8-tetrachlorodibenzo-*p*-dioxini-induced oxidative damage of heart, liver, and kidney tissues has been shown [17]. Besides, dietary capsaicin is capable to alleviate hepatic oxidative stress and apoptosis in rats on high fat diet through balancing oxidant-antioxidant status [18]. Meanwhile, capsaicin can alleviate LPS-induced inflammatory cytokine generation including IL-1β, IL-6 and TNF-α, partly mediated though inhibition of NF-κB [19] and is capable to exert protective effect in the liver and lung tissues against LPS injury that is mediated via appropriate modulation of oxidative status and alleviation of inflammation [20]. Capsaicin can also prevent acute kidney injury through attenuation of mitochondrial dysfunction linked to Nrf2 activation [21]. There is currently no research evidence on the beneficial effect of capsaicin on apoptosis and mitochondrial biogenesis and function in septic models of ALI. Hence, this study was designed and conducted to assess hepatoprotective effect of capsaicin in LPS/D-Gal model of ALI with emphasis on its beneficial effect on attenuation of mitochondrial dysfunction and apoptosis.

## 2 Materials and methods

### 2.1 Animals

Male mice (C57BL/6 strain; n = 40; obtained from Razi Institute of Karaj, Iran) were kept for 1 week for being adapted to animal house conditions. All animals were kept at stipulated conditions (22–23 °C, 42–48% humidity, 12-h lighting photoperiod, and with free access to food and water). All procedures conducted on animals were according to NIH protocols that were approved by NIMAD Institute (IR.NIMAD.REC.1397.163).

### 2.2 Experimental procedures

Animals were randomly divided into 4 testing groups using random number table as follows: control, LPS/D-Gal, and LPS/D-Gal groups receiving capsaicin (Cat #M2028, >95%, SigmaAldrich, USA) at doses of 5 or 20 mg/kg. Mice in LPS/D-Gal group had intraperitoneal injection of a combination of LPS (50 μg/kg) isolated from *E. coli* (Cat #L2630, >95%, SigmaAldrich, USA) and D-Gal hydrochloride (Cat #G0500, >99%, SigmaAldrich, Germany) at a dose of 400 mg/kg (dissolved in normal saline) [22]. Treatment groups received capsaicin (*p.o.* through the gavage needle) daily for 3 days till 1 h before LPS/D-Gal injection. Dose of capsaicin was chosen from its efficacy in amelioration of alcohol-induced ALI [23]. After 6 h, mice were deeply anesthetized with ketamine (150 mg/kg) and after drawing blood samples through the heart were killed and their liver samples were collected for biochemical or histological assessment.

### 2.3 Measurement of serum activity of ALT and AST

Blood samples were drawn from the heart under deep anesthesia with ketamine-HCl. The blood samples were kept at room temperature for 30 min and were then centrifuged at 3000×*g* for 10 min to separate serum samples. Serum activity of ALT (Cat # 1022003, Pars Azmun Co., Tehran, Iran) and AST (Cat # 97203232, Pars Azmun Co., Tehran, Iran) was measured per provided instructions of kits.

### 2.4 Hepatic evaluation of oxidative stress-associated parameters

After preparing liver homogenate samples using 150 mM Tris-HCl buffer (pH 7.4) and centrifuging them, the obtained supernatant was used for measurement of oxidative stress factors. Levels of MDA, which is known as an index of lipid peroxidation, were measured using MDA assay reagent containing trichloroacetic acid (Cat #T4885, >99%, SigmaAldrich, USA) and 2-thiobarbituric acid (Cat #T5500, >98%, SigmaAldrich, USA). Nitrite level as an end-product of nitric oxide (NO) catabolism was evaluated using Griess protocol with its reagent containing sulfanilamide (Cat #S9251, >98%, SigmaAldrich, USA) and N-(1-Naphthyl)ethylenediamine dihydrochloride (Cat # 33461, >98%, SigmaAldrich, USA) in an acidic medium [24]. Activity of SOD was obtained with the help of its specific kit (Cat # 706002, Cayman Chemical, USA). Catalase activity was determined in accordance to Claiborne's method in which disappearance of peroxide was followed spectrophotometrically at 240 nm using potassium phosphate buffer (pH 7) and 0.059 M hydrogen peroxide [25,26]. Bradford method was used for measurement of total protein [27] using its specific kit (Cat # KBRF96, Kiazist, Hamadan, Iran).

### 2.5 Determination of hepatic IL-1β, TNF-α, sirtuin 1, HO-1, IL-10, NF-kB, Nrf2, and TLR4

Liver tissue levels of these factors were determined using sandwich Elisa protocol with antibodies or kits as follows: IL-1β (Cat # RAB0274, SigmaAldrich, USA), TNF-α (Cat # RAB0477, SigmaAldrich, USA), sirtuin 1 (Cat # sc-74465, Santa Cruz Biotechnology, Inc., USA), HO-1 (Cat # sc-390991, Santa Cruz Biotechnology, Inc., USA), IL-10 (Cat # sc-365858, Santa Cruz Biotechnology, Inc., USA), NF-kB (Cat # sc-8008, Santa Cruz Biotechnology, Inc., USA), Nrf2 (Cat # sc-722, Santa Cruz Biotechnology, Inc., USA), and TLR4 (Cat # SAB5700684, SigmaAldrich, USA).

### 2.6 Real-time qPCR

Total RNA was extracted from the liver tissue with Kiazol reagent (Cat # KZOL50, Kiazist, Iran) and reverse transcribed into cDNA with the SYBR Green qPCR Master Mix (Cat # MM2042, Sinaclon, Iran). Forward and reverse primers for the related genes were designed using Primer Express software (Applied Biosystems, USA) and sequences were as follows:

TLR4, CAAGAACATAGATCTGAGCTTCAACCC (forward), GCTGTCCAATAGGGAAGCTTTCTAGAG (Reverse); Bcl2, GACTGAGTACCTGAACCGGCATC (forward), CTGAGCAGCGTCTTCAGAGACA (reverse); Bax, CGAATTGGCGATGAACTGGA (forward), CAAACATG TCAGCTGCCACAC (reverse); NF-kB p65, GAGGCACGAGGCTCCTTTTCT (forward), GTAGCTGCATGGAGACTCGAACA (reverse); PGC-1α, TATGGAGTGACATAGAGTGTGCT (forward), GTCGCTACACCACTTCAATCC (reverse); Nrf2, TTGGCAGAGACATTCCCAT (forward), GCTGCCACCGTCACTGGG (reverse), HO-1, CACGCATATACCCGCTACCT (forward), CCAGAGTGTTCATTCGAGCA (reverse); AMPK, GTCAAAGCCGACCCAATGATA (forward), CGTACACGCAAATAATAGGGGTT (reverse); Sirtuin 1, TGATTGGCACCGATCCTCG (forward), CCACAGCGTCATATCATCCAG (reverse), beta actin, ACTGCCGCATCCTCTTCCT (forward), TCAACGTCACACTTCATGATGGA (reverse). Thermal cycling conditions were denaturation at 95 °C for 10 min, followed by 40 cycles of 95 °C for 15 s and 60 °C for 1 min. Relative differences were expressed using cycle time (Ct) values and data were expressed as a fold change relative to the control and according to 2^-ΔΔCt.

### 2.7 Assessment of apoptosis

For estimation of apoptotic process, liver level of DNA fragmentation (using Cell Death Detection ELISA Plus kit (Cat # 11774425001, Roche, USA) and caspase 3 activity [28] were determined.

### 2.8 Estimation of MMP

MMP as an indicator of mitochondrial integrity was determined according to a previous study with Rhodamine 123 (Cat #R8004, SigmaAldrich, USA) as the detecting probe [29]. In this test, supernatant was re-centrifuged at 10,000 rpm for 15 min and 20 μl of rhodamine 123 solution (10 μmol/L) and 180 μL of PBS was added to the formed precipitate. After stirring, it was transferred to 96-cell microplate and incubated at 37 °C for 30 min. Finally, MMP was determined after excitation at 488 nm and emission at 525 nm.

### 2.9 Histopathology of the liver

Liver sections (a thickness of 5 μm) were stained using H&E routine protocol. Histological changes were assessed in randomly selected microscopic fields at a magnification of 200. Severity of hepatic damage was graded according to a four-point scale from 0 to 3, according to no evidence of damage, moderate to severe damage with widespread nuclear pyknosis, loss of intercellular borders and severe necrosis with hemorrhage and neutrophil infiltration [30]. For TLR4 immunohistochemistry, liver sections were incubated with primary TLR4 antibody (TLR4 (Cat # SAB5700684, SigmaAldrich, USA) and then with secondary HRP-conjugated antibody (Cat # SAB3700852, SigmaAldrich, USA). Every analysis was repeated twice and its data was averaged.

### 2.10 Statistical analysis

All data were expressed as mean ± SEM and statistically analyzed by one-way ANOVA and Tukey tests after verification of parametric distribution of data by Shapiro-Wilk test. Statistical significance was accepted at P less than 0.05.

## 3 Results

### 3.1 The effect of capsaicin on serum activity of ALT and AST

**Fig. 1:**
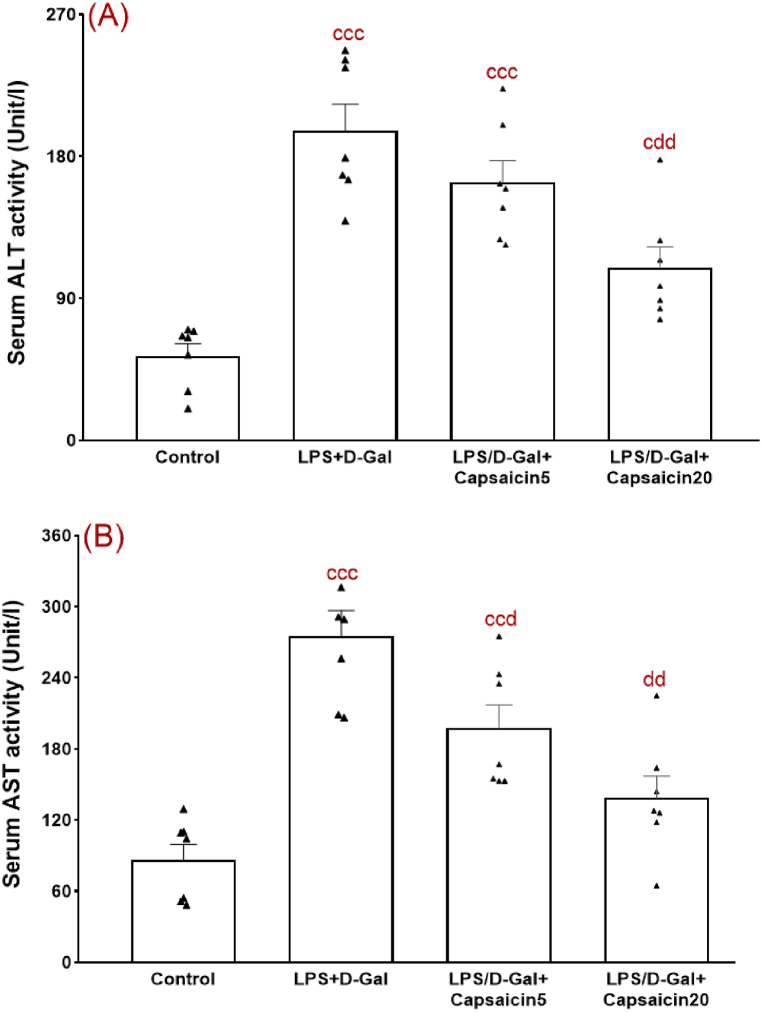
Serum indicators of liver function comprising ALT (A) and AST (B). Data are shown in means ± SEM. Experiments were done in duplicate. ^c^, ^cc^, and ^ccc^ indicate p values lower than 0.05, 0.01, and 0.001, respectively (relative to the control); ^d^ and ^dd^ indicate p values less than 05 and 0.01, respectively (relative to the LPS/D-Gal group). n = 7 per group.

The enzymes ALT and AST are two reliable indicators for estimation of liver function and their notable elevation is the biochemical basis for diagnosing liver damage [31]. The beneficial effect of capsaicin on serum activity of ALT and AST are shown in Fig. 1. Statistical analysis with one-way ANOVA indicated significant inter-group differences regarding ALT (F (3,24) = 22.57, p < 0.001) (Fig. 1A) and AST (F (3,24) = 19.60, p < 0.001) (Fig. 1B). LPS/D-Gal group has a significantly elevated level of the serum activity of these enzymes relative to the control (p < 0.001). In contrast, capsaicin pretreatment at a dose of 20 mg/kg significantly decreased serum activities of ALT (p < 0.01) and AST (p < 0.01) as compared to the LPS/D-Gal-challenged group. Besides, capsaicin at the lower dose of 5 mg/kg did not exert such beneficial effects at a significant level.

### 3.2 The effect of capsaicin on oxidative stress-related factors

**Fig. 2:**
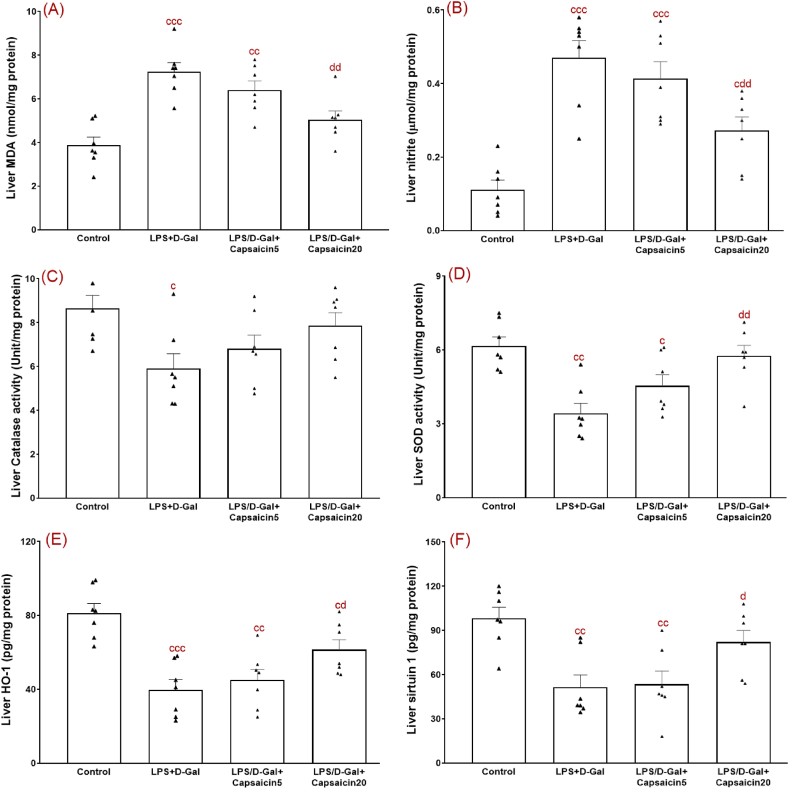
Hepatic levels of oxidative stress-associated indices consisting of MDA (A), nitrite (B), catalase activity (C), SOD activity (D), HO-1 (E), and sirtuin 1 (F). ^c^, ^cc^, and ^ccc^ indicate p values lower than 0.05, 0.01, and 0.001, respectively (relative to the control); ^d^ and ^dd^ indicate p values less than 0.05 and 0.01, respectively (relative to the LPS/D-Gal group). n = 7 per group. Experiments were done in duplicate. All data are presented as mean ± SEM.

LPS/D-Gal challenge increases hepatic oxidative stress that is confirmed by elevated levels of MDA (as the final product of lipid peroxidation process) and ROS and lower levels of some antioxidants [30]. Hence, we explored the effect of capsaicin on the liver levels of some oxidative stress-related indices in this model of ALI. Statistical analysis of oxidative stress data showed significant differences between the group for MDA (F (3,24) = 13.49, p < 0.001), nitrite (F (3,24) = 16.31, p < 0.001), catalase (F (3,24) = 3.68, p < 0.05), SOD (F (3,24) = 9.18, p < 0.001). Mice challenged with LPS/D-Gal had significantly elevated levels of MDA (Fig. 2A) (p < 0.01) and nitrite (Fig. 2B) (p < 0.001) as compared to relevant data of the control group. Our findings also showed significantly lower levels of catalase activity (Fig. 2C) (p < 0.05) and SOD activity (Fig. 2D) (p < 0.01) when compared with comparable findings of the control group. On the contrary, capsaicin pretreatment of LPS/D-Gal group at 20 mg/kg, but not at 5 mg/kg, significantly and suitably lowered level of MDA (p < 0.01) and nitrite (p < 0.01) and improved level of SOD (p < 0.01) and with no significant improvement of the enzyme catalase.

**Fig. 3:**
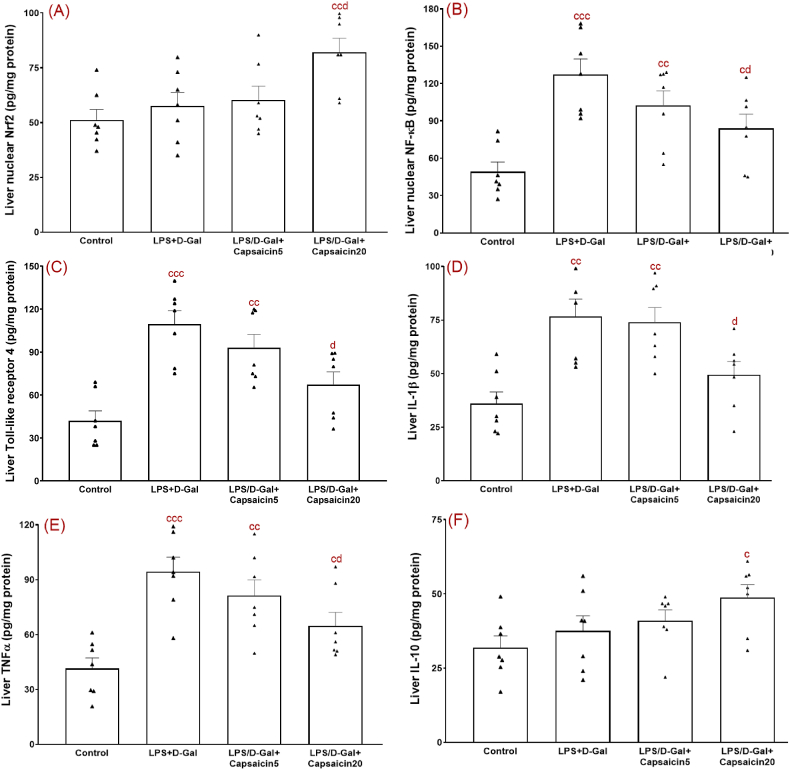
Hepatic levels of Nrf2 (A), NF-kB (B), TLR4 (C), IL-1β (D), TNFα (E), and IL-10 (F). ^c^, ^cc^, and ^ccc^ indicate p values lower than 0.05, 0.01, and 0.001, respectively (relative to the control); ^d^ and ^dd^ indicate p values less than 05 and 0.01, respectively (relative to the LPS/D-Gal group). All data are presented as mean ± SEM. Experiments were done in duplicate. n = 7 per group.

Part of enhancement of antioxidant elements following administration of natural products in models of disorders is mediated through an enhancement of nuclear translocation of Nrf2 [32]. Thus, we also measured nuclear level of Nrf2 (Fig. 3A) and its mRNA gene expression (Table 1). Our results showed no significant change of Nrf2 level or its gene expression in LPS/D-Gal group. On the contrary, there was a significant elevation of Nrf2 (F (3,24) = 5.06, p < 0.01) (p < 0.05) and its gene expression (p < 0.05) due to administration of capsaicin at a dose of 20 mg/kg to LPS/D-Gal group. Nrf2 cascade is associated with HO-1 with critical function in prevention of oxidative stress and inflammation. In this study, HO-1 level (Fig. 2E) (F (3,24) = 11.82, p < 0.01) (p < 0.01) and its gene expression (Table 2) (p < 0.01) was significantly lower in LPS/D-Gal-injured group versus the control group. Conversely, capsaicin at 20 mg/kg significantly increased HO-1 level (p < 0.05) and its gene expression (p < 0.05) versus the vehicle-treated injured group.

**Table 1:** The effect of capsaicin on mitochondrial and apoptotic indices in the liver tissue following LPS/D-Gal-induced hepatotoxicity.

	Control	LPS/D-Gal	LPS/D-Gal + Capsaicin5	LPS/D-Gal + Capsaicin20
**Apoptosis**				
DNA fragmentation	0.37 ± 0.05	0.89 ± 0.08^ccc^	0.82 ± 0.09^cc^	0.57 ± 0.08^d^
Caspase 3 activity	0.38 ± 0.04	0.92 ± 0.07^ccc^	0.94 ± 0.08^ccc^	0.63 ± 0.05^cd^
**Mitochondrial dysfunction**				
MMP (AFU)	100 ± 0.00	53.8 ± 4.7	57.1 ± 5.4	74.2 ± 5.1^d^

Data are shown as means ± SE. ^c^, ^cc^, and ^ccc^ suggest p < 0.05, 0.01, and 0.001, respectively (as compared to the control); ^d^ suggest p < 05 (as compared to the LPS/D-Gal group). AFU indicates arbitrary fluorescence unit. n = 7 per experimental group. Experiments for DNA fragmentation and caspase 3 were done in duplicate and for MMP were conducted once.

**Table 2:** The effect of capsaicin on gene expression of ΑΜPK, Bax, Bcl2, PGC-1α, sirtuin 1, HO-1, NF-κB, TLR4, and Nrf2 in the liver tissue following LPS/D-Gal-induced hepatotoxicity.

	Control	LPS/D-Gal	LPS/D-Gal + Capsaicin5	LPS/D-Gal + Capsaicin20
**Relative mRNA expression**				
Bax	0.27 ± 0.03	0.98 ± 0.12^ccc^	0.75 ± 0.10^c^	0.55 ± 0.09^d^
Bcl2	1.14 ± 0.04	0.53 ± 0.09^cc^	0.61 ± 0.10^cc^	0.92 ± 0.09^d^
TLR4	0.57 ± 0.04	1.37 ± 0.10^ccc^	1.15 ± 0.11^cc^	0.97 ± 0.08^cd^
NF-κB	0.68 ± 0.05	1.49 ± 0.12^ccc^	1.25 ± 0.13^c^	0.91 ± 0.10^dd^
Nrf2	1.09 ± 0.04	1.27 ± 0.08	1.42 ± 0.10^c^	1.63 ± 0.08^ccd^
HO-1	1.17 ± 0.05	0.64 ± 0.11^cc^	0.73 ± 0.09^c^	1.03 ± 0.08^d^
PGC-1α	0.95 ± 0.04	0.41 ± 0.09^cc^	0.53 ± 0.08^cc^	0.75 ± 0.08^d^
Sirtuin 1	1.73 ± 0.05	0.85 ± 0.12^ccc^	1.14 ± 0.13^c^	1.59 ± 0.12^dd^
AMPK	1.19 ± 0.06	0.94 ± 0.11	1.27 ± 0.14	1.48 ± 0.12^d^

Data are shown in means ± SEM. ^c^, ^cc^, and ^ccc^ indicate p values lower than 0.05, 0.01, and 0.001, respectively (relative to the control); ^d^ and ^dd^ indicate p values less than 05 and 0.01, respectively (relative to the LPS/D-Gal group). n = 7 per experimental group.

There is a crosstalk between oxidative stress, sirtuin 1, and inflammation. In this regard, during oxidative and inflammatory events, level of sirtuin 1 decreases and vice versa [33]. Our results showed significant and marked fall of sirtuin 1 level (Fig. 2F) (F (3,24) = 7.84, p < 0.001) (p < 0.01) and its gene expression (Table 2) (p < 0.001). In contrast, giving capsaicin orally at a dose of 20 mg/kg, but not at the lower dose of 5 mg/kg, was capable to properly and significantly elevate hepatic level of sirtuin 1 (p < 0.05) and also its gene expression (p < 0.01).

### 3.3 The effect of capsaicin treatment on inflammation-associated factors

To properly assess involvement of inflammation following LPS/D-Gal challenge and to evaluate capsaicin efficacy, we measured liver level of nuclear level of NF-kB (Fig. 3B) and its mRNA gene expression (Table 2), level of TLR4 (Fig. 3C) and its gene expression (Table 2), (IL-1β) (Fig. 3D), TNF-α (Fig. 3E) and IL-10 (Fig. 3F). Our obtained data showed elevated levels of NF-kB (F (3,24) = 8.92) (p < 0.001) and its gene expression (p < 0.001), TLR4 (F (3,24) = 11.57) (p < 0.001) and its gene expression (p < 0.001), IL-1β (F (3,24) = 8.53) (P < 0.01), and TNF-α (F (3,24) = 9.25) (p < 0.001) besides non-significant and slight increase of anti-inflammatory factor IL-10 β (F (3,24) = 2.77) in the LPS/D-Gal group. In contrast, capsaicin pretreatment of LPS/D-Gal-challenged group at a dose of 20 mg/kg, but not at a dosage of 5 mg/kg, reduced hepatic levels of NF-kB (p < 0.05) and its gene expression (p < 0.01), TLR4 (p < 0.05) and its gene expression (p < 0.05), IL-1β (p < 0.05), and TNF-α (p < 0.01) and properly elevated hepatic level of IL-10 (p < 0.05) when these findings are compared with the LPS/D-Gal group.

### 3.4 The effect of capsaicin treatment on hepatic apoptosis-associated factors

We take into account caspase 3 activity and DNA fragmentation (Table 1) as known apoptotic indices [34]. Statistical analysis of apoptosis-associated data indicated significant differences between the groups for caspase 3 (F (3,24) = 11.75, p < 0.001) and DNA fragmentation (F (3,24) = 10.38, p < 0.001). The LPS/D-Gal group had a significantly elevated activity of caspase 3 (p < 0.001) and also higher level of DNA fragmentation (p < 0.001) versus the control group. On the contrary, capsaicin administration to LPS/D-Gal-challenged group at a dose of 20 mg/kg significantly and suitably reduced DNA fragmentation (p < 0.05) and also activity of caspase 3 (p < 0.05) when comparing these findings with relevant data of the LPS/D-Gal group. To further evaluate the beneficial effect of capsaicin on apoptosis at gene expression level, we determined liver mRNA for pro-apoptotic factor Bax and anti-apoptotic factor Bcl2, as shown in Table 2. Analysis of data showed significantly higher gene expression for Bax (p < 0.001) and lower gene expression for Bcl2 (p < 0.01) in LPS/D-Gal group. In addition, capsaicin given at a dose of 20 mg/kg significantly reduced Bax gene expression (p < 0.05) and raised Bcl2 gene expression (p < 0.05), indirectly indicating lower rate of apoptosis in the liver tissue.

### 3.5 The effect of capsaicin on mitochondrial homeostasis and biogenesis

Development of mitochondrial dysfunction in disease conditions leads to liver injury [35]. During a septic insult, mitochondrial biogenesis is upset which is indicated by lower expression for PGC-1α as its key regulator [36]. In this study, to have an evaluation of mitochondrial biogenesis and health status, we measured MMP (Table 1) in addition to determination of gene expression for PGC-1α and AMPK (Table 2). Analysis of data showed lower level of MMP (F (3,24) = 9.83) (p < 0.01) besides reduction of PGC-1α (p < 0.01) and AMPK (p > 0.05) in the LPS/D-Gal group as compared to the control group. Contrarily, capsaicin at a dose of 20 mg/kg appropriately prevented MMP fall (p < 0.05) and PGC-1α (p < 0.05) and AMPK (p < 0.05) reduction.

### 3.6 The effect of capsaicin on liver histopathology

**Fig. 4:**
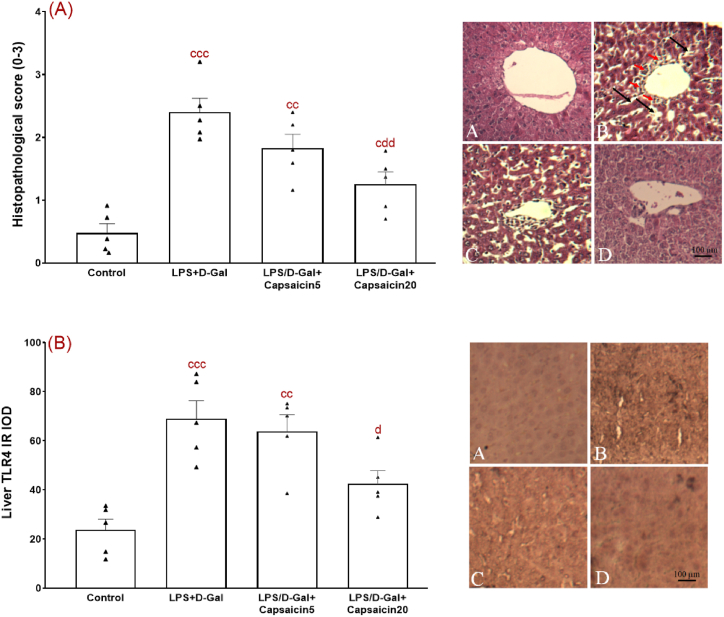
Severity of histopathological changes (on the basis of a 0–3 scoring) and typical histopathologic alterations of the liver tissue sections stained with H&E protocol (A) and TLR4 immunoreactivity (IRA) using immunohistochemistry method (B). (A) Control, (B) LPS/D-Gal, (C) LPS/D-Gal + Capsaicin at a dose of 5 mg/kg, and (D) LPS/D-Gal + Capsaicin at a dose of 20 mg/kg n = 5 per group. Solid red arrows indicate infiltration of defensive cells and solid black arrows show derangement of liver cell organization with abnormal and expanded sinusoidal spaces and/or cytoarchitectural disarrangement. Experiments were done once and every analysis was repeated twice and its data was averaged for each specimen. (For interpretation of the references to colour in this figure legend, the reader is referred to the Web version of this article.)

Fig. 4A shows histopathological results based on a 0–3 scoring according to severity of injury in different groups and typical photomicrographs of the liver tissue. Microscopic assessment of liver tissue from the control group showed normal structure such as clear central vein, distinct hepatocytes, and normal sinusoidal spaces. On the contrary, liver tissue of LPS/D-Gal group had robust pathologic changes, as shown by widespread necrotic areas, neutrophil infiltration and presence of inflammatory cells and even derangement of liver cell organization as compared with the control group. Accordingly, severity of pathological score for LPS/D-Gal group was significantly higher when compared to the control group (p < 0.001). These inappropriate changes were less evident in capsaicin-pretreated LPS/D-Gal groups, so pathological score in LPS/D-Gal + capsaicin20 group (but not LPS/D-Gal + capsaicin5 group) was significantly lower than LPS/D-Gal group (p < 0.01) (F (3,16) = 17.32, p < 0.001).

Evaluation of TLR4 immunoreactivity in our experimental groups indicated a significant inter-group differences (F (3,16) = 11.69, p < 0.001) (Fig. 4B). Further Tukey post-test showed a markedly higher TLR4 IRA in LPS/D-Gal group versus the control group (p < 0.001). Conversely, this immunoreactivity for TLR4 was significantly lower in capsaicin20-treated LPS/D-Gal group when compared to vehicle-treated LPS/D-Gal group (p < 0.05).

## 4 Discussion

This research study was conducted to show possible hepatoprotective effect of capsaicin in LPS/D-Gal model of ALI. Administration of capsaicin at the higher dose (20 mg/kg) to LPS/D-Gal-injured animals decreased liver functional markers besides its reduction of oxidative stress, inflammation, apoptosis, and mitochondrial dysfunction.

Liver is a vital tissue for the detoxification of toxic substances and excessive exposure to toxicants is associated with hepatic injury [37]. Liver damage due to a challenge of LPS/D-Gal in rodents is regarded a reliable model to test the possible efficacy of new drugs, especially natural products, to prevent ultimate liver failure [38]. To ameliorate sepsis-induced tissue injury, inhibition of inflammatory and oxidative stress factors is of paramount significance [39]. To manage and treat disorders of liver and its associated complications, research studies have emphasized on finding of novel agents with antioxidant and anti-inflammatory potential. Hence, we selected capsaicin as the main effective ingredient of red chili to explore its possible efficacy in LPS/D-Gal model of ALI.

The activity of aminotransferase enzymes ALT and AST is taken as reliable and consistent indicators of liver function. ALT and AST changes indicate somehow hepatocellular dysfunction. Hence, blood levels of these enzymes increase following liver diseases [40]. Similarly, in this study, serum activities of AST and ALT significantly were higher after LPS/D-GAL. On the contrary, capsaicin administration given at 20 mg/kg ameliorated these changes which is indicative of its suppression of hepatic disturbance. In agreement with our finding, it has been shown that capsaicin has hepatoprotective effect in mice on a high-fat diet, as shown by lower levels of ALT and AST, that is exerted partly through its alleviation of mitochondrial oxidative stress and improvement of mitochondrial function and bioenergetics [41].

Furthermore, we also showed protective potential of capsaicin on the liver tissue, as demonstrated by its amelioration of hemorrhagic areas, necrosis, neutrophil infiltration and prevention of liver cell disarrangement. In line with this finding, it has been shown that capsaicin is capable to exert a protective effect in the liver tissue in carbon tetrachloride (CCl4) model of hepatotoxicity in the rat [42]. Additionally, other studies have also recently shown protective effect of capsaicin in other tissues under toxic conditions that is partly due to its antioxidant and anti-inflammatory potential [21,43].

Earlier studies have shown the involvement of oxidative stress in LPS/D-Gal-induced ALI [11]. Obtained findings of this study showed significant elevation of MDA and nitrite and reduction of catalase and SOD activity following LPS-D-Gal that is in agreement with past studies [44,45]. On the contrary, capsaicin alleviated oxidative stress, as was shown by reversing some of these changes. To support our obtained data, it has been shown that capsaicin due to its anti-oxidative potential is capable to exert a hepatoprotective effect against concanavalin A through suppressing oxidative stress and inflammation [16]. In another study, it was shown that capsaicin could ameliorate alcohol-induced ALI, partly via improvement of hepatic antioxidant status [23]. In addition, Nrf2 is known as an important redox-sensing transcription factor that governs the gene expression of endogenous antioxidants [46]. Nrf2 itself control and induces the expression of HO-1 with important roles in cellular antioxidant axis [47]. Studies have shown that activation of Nrf2/HO-1 pathway produces a protective effect in LPS/D-Gal model of liver injury [48]. In this study, capsaicin administration significantly improved level and gene expression of Nrf2 in LPS/D-Gal group which is responsible for part of its anti-oxidative potential. In agreement with this finding, it has been shown that capsaicin can prevent contrast-associated kidney damage, partly via activation of Nrf2 cascade [21].

Sirtuin 1 is a nicotinamide adenine dinucleotide (NAD)-associated deacetylase that plays important tasks in prevention of apoptosis, DNA injury, and imbalance of mitochondrial metabolism [49]. Sirtuin 1 protects living cells against oxidative injury via Nrf2 pathway [50] and its upregulation attenuates inflammatory events through suppression of NF-κB cascade [51]. Hence, sirtuin 1 upregulation can mitigate oxidative and inflammatory events [52]. In addition, down-regulation of sirtuin 1 in the liver tissue has been reported following LPS/D-Gal [53]. In our study, capsaicin was capable to raise gene expression of sirtuin 1 besides preventing reduction of its tissue level. In line with this finding, capsaicin can alleviate intermittent high glucose-induced endothelial senescence via elevating sirtuin 1 and proper regulation of TRPV1/AMPK pathways [54].

LPS/D-Gal challenge is associated with activation of Kupffer cells with ensuing generation and release of inflammatory cytokines encompassing IL-1β, IL-6, and TNFα [55]. In this study, LPS/D-Gal injection caused significant elevation of inflammatory factors besides elevation of NF-kB and TLR4, clearly denoting the occurrence of inflammation in the liver. Contrarily, capsaicin administration alleviated inflammation severity, as was apparent by lower quantities of proinflammatory cytokines. Anti-inflammatory potential of capsaicin in acetaminophen-induced ALI has been reported before [56].

LPS/D-Gal also increases hepatic apoptosis, as shown by higher rate of DNA fragmentation and higher activity of caspase 3 [57] that was also shown in this study. In contrast, capsaicin was able to significantly alleviate hepatic levels of these apoptotic markers that is also confirmed in earlier studies [18]. In addition, capsaicin was capable to reduce gene expression of pro-apoptotic factor Bax and to elevate gene expression for anti-apoptotic factor Bcl2 and theses alterations produce lower level of apoptosis in the liver tissue. Of relevance to our findings on apoptosis, capsaicin can protect against LPS-induced acute lung injury via down-regulation of caspase 3 and Bax expression and up-regulation of Bcl-2 besides its proper regulation of NF-κB/PI3K/AKT/mTOR cascades [43].

Changes in mitochondrial metabolism and biogenesis play a pivotal role in different diseases [58]. Past studies have indicated that mitochondrial dysfunction is a key factor in the development of ALI [59,60]. A challenge of LPS is associated with disturbance of mitochondrial dynamics and biogenesis in the liver [61]. Expression or level of PGC-1α (PPARγ coactivator-1α) as a key regulator of mitochondrial function decreases during LPS-induced ALI [62]. In this study, LPS/D-Gal-provoked ALI reduced MMP in addition to down-regulation of PGC-1α and its gene expression which has also been reported separately in earlier studies [63,64]. Capsaicin pretreatment significantly improved dysfunction of mitochondrial function, as indicated by higher level of MMP and greater level and gene expression of PGC-1α. In agreement with this finding, previous studies have shown that capsaicin can protect cardiomyocytes against LPS injury via improvement of mitochondrial function [65]. In addition, capsaicin affects lipogenesis in HepG2 cells via activating and/or up-regulating AMPK/PGC-1α [66]. Besides PGC-1α and sirtuin 1 as the central regulators of mitochondrial biogenesis, AMPK signaling also plays a pivotal role in regulation of mitochondrial biogenesis, inflammation, and apoptosis and it is essential for maintenance of cell homeostasis [67]. In the current study, capsaicin properly elevated gene expression of AMPK following LPS/D-Gal challenge. In support of our finding, it has been shown that part of protective effect of capsaicin in LPS model of cardiomyocyte damage is through regulation of AMPK/mTOR pathways and in this way can inhibit oxidative stress and inflammation as well as its maintenance of mitochondrial function and autophagy augmentation [65].

Lack of further histochemical studies including NF-kB and Nrf2 immunohistochemistry and absence of Western blotting experiments were some limitations of the present study which may be taken into account in future relevant studies.

To conclude, this study indicated hepatoprotective property of capsaicin under septic conditions that can be attributed to its down-regulation of oxidative and inflammatory processes besides its potential to attenuate mitochondrial dysfunction and apoptosis. This maybe of potential benefit in clinical settings after further studies.

## Author contribution statement

Tourandokht Baluchnejadmojarad and Mehrdad Roghani conceived and designed the study, analyzed and interpreted the data, and wrote the paper; Atefeh Ghorbanpour and Sepide Salari performed the experiments, analyzed and interpreted the data, and wrote the paper.

## Funding statement

This research project was financially supported in 2018 by National Institute for Medical Research Development (NIMAD) of Iran (grant number 965431).

## Data availability statement

Data sets of this study are available from the corresponding author on reasonable request.

## Conflict of interest statement

The authors declare no conflict of interest.

## Additional information

No additional information is available for this paper.
